# Hematopoietic Stem and Progenitor Cells in Inflammation and Allergy

**DOI:** 10.3389/fimmu.2013.00428

**Published:** 2013-12-04

**Authors:** Kimberly D. Fischer, Devendra K. Agrawal

**Affiliations:** ^1^Department of Medical Microbiology and Immunology, Creighton University School of Medicine, Omaha, NE, USA; ^2^Department of Biomedical Sciences, Creighton University School of Medicine, Omaha, NE, USA; ^3^Department of Internal Medicine, Creighton University School of Medicine, Omaha, NE, USA; ^4^Center for Clinical and Translational Science, Creighton University School of Medicine, Omaha, NE, USA

**Keywords:** allergic asthma, hematopoietic stem/progenitor cells, inflammation, eosinophils, fibrocytes

## Abstract

Hematopoietic stem and progenitor cells contribute to allergic inflammation. Pro-inflammatory cytokines that are generated following allergen challenge can impact the differentiation of hematopoietic progenitor cells leading to increased production of effector cells such as eosinophils and basophils, which are key cells involved in the pathogenesis of allergic airway inflammation. Homing of stem cells to the lungs is associated with inflammatory and remodeling changes in asthmatics. Factors that modulate the differentiation and increased migration of stem cells to the site of inflammation in asthma remain to be defined. Stem cells can mature at the site of inflammation in response to inflammatory mediators and other components in the milieu. While the available data suggest that hematopoietic cells traffic to target tissues, the molecular factors underlying *in situ* differentiation have yet to be specified. Here, we critically evaluate the potential role of hematopoietic progenitors in contributing to the increased immune cell infiltrate in allergic asthma and the factors that drive their differentiation.

## Introduction

Asthma is a chronic inflammatory disease of the airways characterized by infiltration of immune cells, hypersensitivity, bronchoconstriction, airway obstruction, and airway remodeling (1). One of the most common types of asthma is allergic asthma. Upon initial exposure to allergens, the antigen is phagocytized by mucosal dendritic cells (DCs), which will then travel to regional draining lymph nodes and present the antigen to naive helper T cells (Th0). The mechanisms underlying the differentiation of Th0 to type-2 helper T (Th2) cells, the predominant cells found in allergic airway inflammation, are not well understood. It is hypothesized that the environment of the lymph node as well as cytokines released by activated DCs, cause the differentiation into a Th2 cell (2, 3). The Th2 cells then travel back to the bronchial mucosa where they can drive class switching and IgE production by plasma cells at the site of allergic inflammation through secretion of IL-4, IL-5, and IL-13 (4). IgE binds primarily to the Fcε receptor I (FcεRI) on mast cells and basophils in the airway. This sensitization event primes the airway tissue, so that upon secondary exposure to an allergen, receptor cross-linking on mast cells and basophils causes release of their cellular contents consisting of major basic protein, histamine, prostaglandins, and leukotrienes, which cause bronchoconstriction and epithelial cell damage. Chemokines released by these cells recruit other immune cells, including macrophages and neutrophils, which release their granular products to further exaggerate inflammation and tissue damage (3, 5, 6).

Differentiation of hematopoietic stem/progenitor (HSPCs) into different immune cells in the presence of locally elevated cytokines within the lung tissue can provide a continuing source of inflammation and change in structural cells (7, 8). Allergen-induced increases in CD34^+^ HSPCs in the bone marrow and airways suggest that a component of the pathophysiology of allergic asthma involves trafficking of hematopoietic stem cells from the bone marrow to the lung. The observation that progenitor cells are responsive to specific cytokines prompted us to look at the differentiation and migration of stem cells and their contribution in allergic asthma. The focus of this review is to evaluate components of HSPCs cell differentiation and the identification of these cells in the promotion and amplification of the allergic asthmatic response in the lung. We will also address the potential therapeutic targets in controlling differentiation and homing of hematopoietic stem and progenitor cells.

## Hematopoietic Stem Cells

Hematopoietic progenitors are defined as undifferentiated pluripotent stem cells capable of self-renewal and differentiation into all blood cell types. Hematopoietic stem cells circulate in the bloodstream under steady-state conditions (9). CD34, the marker of hematopoietic stem cells, is highly expressed on the progenitor cells, decreases with cell maturation, and is lost fully on mature cells (10). CD45 is also expressed on hematopoietic cells and assists in bone marrow egress and tissue migration (11, 12).

Stem/progenitor cells have been isolated and many studies have defined the role of HSPCs in allergic diseases. During hematopoiesis, hematopoietic stem cells differentiate into multipotent progenitor cells expressing CD34. The multipotent progenitor cells can further differentiate into the common lymphoid progenitor or the common myeloid progenitor. Both of these common progenitor cell types further differentiate into downstream progenitor cell types before finally differentiating into terminally differentiated blood cell types. While most studies utilize CD34 expressing cells as HSPCs-derived cells, it is important to realize that CD34 is expressed on a number of different progenitor cells in the hematopoietic lineage in addition to hematopoietic stem cells (13). The majority of studies investigating the migration of hematopoietic stem cell do not distinguish between hematopoietic stem cells or hematopoietic progenitor cells (Table 1). Therefore, in the following discussion these cells will be referred to as a combined cell population of HSPCs.

**Table 1:** **Classification and characteristics of stem/progenitor cell markers: reports examining the homing and differentiation of HSPCs utilize different markers and starting source materials**.

Stem/progenitor markers	Source	Reference
Forward scatter low, side scatter low, CD34^+^/CD45^dull^	Human cord blood and peripheral blood	Punia et al. (8)
Sca-1^+^, c-kit^+^	Human bone marrow and peripheral blood	Doyle et al. (14)
Side scatter low, CD34^+^/CD45^dull^	Human bone marrow	Sehmi et al. (15)
Monocyte depleted, CCR3^+^ CD34^+^	Human cord blood	Lamkhioued et al. (16)
CD45.2^+^, Lin^−^, c-kit^+^	Mouse thoracic duct and bone marrow	Massberg, et al. (17)
CD34^+^, CD3^−^, CD10^−^, CD14^−^, CD19^−^, CD20^−^, CD40^−^, CD56^−^, CD83^−^, IL-5R^−^, FCεRI^−^	Human peripheral blood and sputum	Allakhverdi et al. (9)
CD34^+^/CD45^+^	Mouse BAL and lung tissue	Southam et al. (18)
Lin^–^Sca-1^+^c-kit^+^	Mouse bone marrow	Dyer et al. (19)
CD34^+^, hCD3^−^, hCD4^−^, hCD8^−^, hCD19^−^, hCD56^−^, CD38^−^	Human cord blood	Takagi et al. (20)

Differentiation of hematopoietic stem cells proceeds in the bone marrow under the influence of resident stromal cells and growth factors to which stem cells are responsive. Hematopoietic stem cells or hematopoietic progenitor cells can leave the bone marrow niche as a requirement for recruitment in response to stromal cell-derived factor 1α (SDF-1α) (21).

The receptors important in the transmigration of HSPCs are C–X–C chemokine receptor type 4 (CXCR4) and C–C chemokine receptor type 3 (CCR3). CXCR4 is a chemokine receptor for SDF-1α (CXCL12). CXCR4 is expressed on hematopoietic and lymphopoietic cells and responds to SDF-1α, which is expressed in the bone marrow, lungs, liver, and lymph nodes. CCR3 is highly expressed on eosinophils and basophils, but is also expressed on T-helper cells, mast cells, airway epithelial cells, as well as in progenitor cells (Table 2). This receptor binds and responds to a variety of chemokines, including eotaxin (CCL11), eotaxin-3 (CCL26), MCP-3 (CCL7), MCP-4 (CCL13), and regulated on activation, normal T cell expressed and secreted (RANTES or CCL5) (22). Other ligands expressed on HSPCs include P-selectin glycoprotein ligand 1 (PSGL-1) and very late antigen-4 (VLA-4) which bind to P-selectin and VCAM-1 located on endothelial cells at the site of entry, respectively (23) (Table 2). While these factors are important for homing to the bone marrow, as previously reviewed (24, 25), the following evidence suggests the involvement of HSPCs in the migration to sites of inflammation using the same cytokines, chemokines, and growth factors.

**Table 2:** **List of cell types and the presence of receptors and their ligands**.

Cell type	Receptor	Ligand
Hematopoietic stem cells	CXCR4	SDF-1α (CXCL12)
Lymphocytes	
Eosinophils		Eotaxin (CCL11)
Basophils		Eotaxin-3 (CCL26)
T-helper cells	CCR3	MCP-3 (CCL7)
Progenitor cells		MCP-4 (CCL13)
		RANTES (CCL5)
Hematopoietic stem cells	c-Kit	SCF
Leukocytes	E-selectin	PSGL
Endothelial cells		Sialylated carbohydrates
Leukocytes	P-selectin	PSGL
Endothelial cells		

## HSPCs in Allergy and Asthma

Growth factors and pro-inflammatory cytokines contribute to the differentiation of HSPCs in allergic diseases, including asthma, and can occur in both the bone marrow and locally at the site of inflammation (9, 15, 18). The importance of hematopoietic stem cells in the initiation and onset of allergy and asthma was demonstrated when bone marrow cells from an allergic donor were transferred to a recipient with no known allergies. This resulted in the recipient with the synthesis of serum IgE levels specific to cat and dog allergens at 29 days post-transplant (26). By 37 months post-transplant, exposure to cat and dog allergens triggered asthma in the recipient. While the underlying mechanism for this case is unknown, it does highlight and support the thesis that hematopoietic cells are likely involved in the development of allergic diseases.

There is an additional evidence for the contribution of HSPCs in asthma that was presented by Doyle and colleagues (14). Here, the authors demonstrated that mice challenged with ovalbumin (OVA) resulted in amplification of hematopoietic stem cells in the airway. Use of a CXCR4 antagonist attenuated hematopoietic stem cell accumulation in the airway and was linked with decreased AHR and airway remodeling (14). In humans, CD34^+^ progenitor cells were increased in the sputum of asthmatic patients following exposure to inhaled allergen. This correlated well with angiogenesis in the bronchial mucosa (27). These findings indicate a role for HSPCs in the inflammatory and remodeling components of asthma.

**Figure 1:**
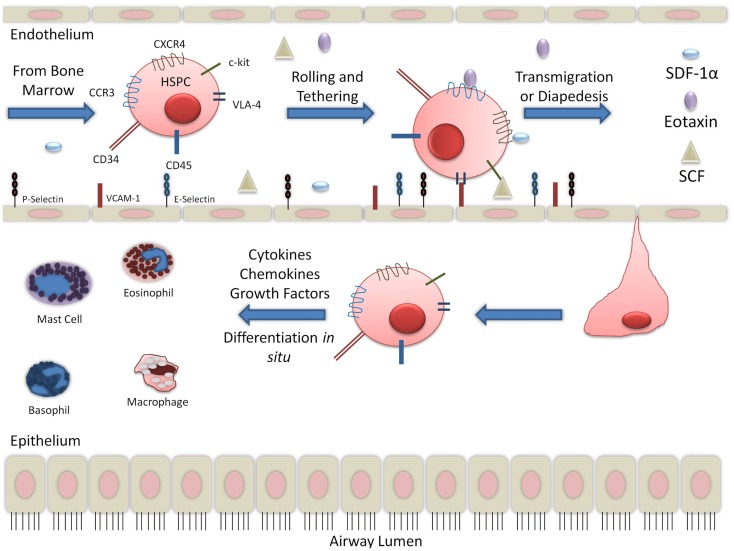
**Migration of hematopoietic progenitor cells into lung tissue: while the migration and diapedesis of hematopoietic stem/progenitor cells are not completely understood, chemoattractants such as eotaxin, SCF, and SDF-1α can initiate HSPC migration to the site of inflammation in the lung tissue**.

There is large body of evidence to suggest that in the context of allergic inflammation, HSPCs have increased migratory capabilities. Stimulation of CD34^+^CD45^+^ cells with SDF-1α and eotaxin increased migratory response in post-allergen stimulation compared to pre-allergen levels. This was correlated with increased expression of CXCR4 and CCR3 (15, 28). CCR3 expression on CD34^+^ cells has been found to be upregulated in the presence of interleukin-4 (IL-4) and IL-5 (16). SDF-1α, with the contribution of Th2 cytokines, increased hematopoietic progenitor cells homing to sites of allergic inflammation (8). SDF-1α is released in the bronchoalveolar lavage (BAL) fluid and has been detected by immunohistochemistry in endobronchial biopsies of asthmatics (29). In asthma exacerbations, SDF-1α was found to be down-regulated in the bone marrow and corresponded with increased expression in asthmatic airways (30). Another chemokine, SCF, was also found to be increased in the serum of non-severe and severe asthmatics (31). Trafficking receptors expressed on HSPCs, including CXCR4, CCR3, and c-kit, likely contribute to the migration of HSPCs to the sites of inflammation since serum from asthmatics contain the chemoattractants, SDF-1α, eotaxin, and SCF, respectively. In asthmatic lung, additional cytokines, including granulocyte macrophage-colony stimulating factor (GM-CSF) and growth factors, released by several cells, including T-helper cells, are found. This suggests that inflammation during an allergic event may promote HSPCs to the lungs, thereby contributing to asthma pathophysiology (Figure 1).

Hematopoietic stem/progenitors have been demonstrated to traffic to different tissues such as lung, liver, and kidney where they reside for several days (17). The migration of HSPCs from the bone marrow may depend on the peripheral environment. CD34^+^ cells isolated from cord and peripheral blood showed increased migratory response in the presence of IL-4. The priming effect of cytokines was found to be through increased incorporation of the SDF-1α receptor CXCR4 into lipid rafts (8). VLA-4 on bone marrow-derived CD34^+^45^+^ cells was found to be decreased following allergen challenge. This may be a factor in facilitating movement of these cells out of the bone marrow and into the peripheral circulation during an inflammatory response (32). Indeed, release of SDF-1α and matrix metalloproteinase from the bone does also occur (33). This suggests that while the cellular signals imposing HSPCs migration to the tissue are indeed similar, changes in either the bone marrow, tissues, or both, dictate the movement of HSPCs. Further studies are warranted to address the parameters that determine when this occurs and what factors are involved.

## HSPC Differentiation into Eosinophils and Basophils

Cytokines and growth factors exert their effects via binding to specific receptors on the cell surface. Stem cell factor (SCF) and IL-6 act early on in the survival and self-renewal of hematopoietic stem cells, while other growth factors, such as IL-5, are largely responsible for the terminal differentiation into eosinophils and basophils (30).

Once within the lung, hematopoietic progenitor cells have the potential to differentiate *in situ*. During asthma exacerbation, the bone marrow is activated and HSPCs traffic to the lung. Cytokines generated following allergen challenge can impact the differentiation and mobilization of HSPCs leading to accelerated eosinophil and basophil production, thus contributing to the ongoing recruitment of pro-inflammatory cells to target tissues sites in allergic diseases. Eosinophil and basophil progenitors are found to be upregulated in peripheral blood, bone marrow, lung tissue, and sputum in response to allergic stimuli, as a result of increased differentiation and migration. The commitment of bone marrow progenitors to the eosinophil and basophil lineage has been demonstrated to be regulated by pro-inflammatory cytokines, including IL-3, IL-5, and GM-CSF, that are commonly found in allergic tissue (30).

Increased eosinophil and basophil progenitors are found to be correlated with increased acute respiratory symptoms in the cord blood of infants (34). Assessment of eosinophil and basophil progenitor cells in peripheral blood from children 1 year of age was performed by using methylcellulose assays, which defines progenitors on their ability to generate colony forming units (CFUs). IL-13 and GM-CSF-responsive eosinophil and basophil CFUs in the blood of these children were found to be associated with exposure to smoking related volatile organic compounds when exposed during pregnancy or first year of life (35). This suggests that these cells could respond to growth factors and cytokines at the site of inflammation. In mice, Starkey and colleagues (36) found that *Chlamydia muridarum* infection of infant mice had altered hematopoietic cells with increased severity in airway hyperresponsiveness during airway allergic disease in later life. Infant mice had increased IL-5 and IL-13 cytokines released from mediastinal lymph node cells along with increased mucus secreting cells, increased transpulmonary resistance, and IL-13 production in the lung (36). In humans, stimulation of cord blood with lipopolysaccharide (LPS) resulted in increased eosinophil and basophil progenitors. This process was mediated by GM-CSF secretion by CD34^+^ cells and required p38 mitogen-activated protein kinases (MAPK) activation (37). These studies further demonstrate the capability of inflammatory events to initiate HPSC differentiation.

Epithelial cell-associated cytokines, such as IL-25 and IL-33, have been suggested to be important in the initiation of allergic responses. IL-25 has been shown to initiate Th2-type airway inflammation but has also been shown to induce multipotent progenitor cells into macrophages, basophils, and mast cells in gut associated lymphoid tissue (2, 38). In IL-25^−/−^ mice, IL-25 was produced by epithelial cells in OVA-challenged mice suggesting that the allergic airway inflammation induced by OVA challenge can initiate IL-25 expression (39). IL-33 was found to be important in the differentiation of HSPCs into eosinophils. IL-33 activity in the bone marrow compartment in mice acted locally on hematopoietic progenitor cells in an IL-5-dependent manner. IL-33 also triggered the production of IL-13 and IL-6, and increased the production of CCL17 and transforming growth factor-β (TGF-β) from wild-type eosinophils. In addition to differentiation of hematopoietic progenitor cells into eosinophils, IL-33 also increased lung levels of eosinophils, macrophages, lymphocytes, IL-13, TGF-β, CCL3, CCL17, and CCL24 in eosinophil-mediated airway inflammation (40). CD34^+^ progenitor cells in humans express a functional receptor for IL-33 that results in the rapid release of high levels of cytokines and chemokines in the presence of IL-33, further contributing to the possibility of IL-33-mediated differentiation of progenitor cells (9). These studies suggest the importance of IL-33 in mediating the differentiation of HSPCs into eosinophils during an allergic event in the airways. Despite these findings, careful investigations are required to confirm IL-25 involvement in HSPC differentiation. Further studies looking at other epithelial cell-derived cytokines, such as TSLP, should also be performed to further elucidate these mechanisms.

Bone marrow progenitors from inbred Rocky Mountain White (IRW) mice were found to be incapable of proliferation or appropriate differentiation in response to SCF, Flt3-ligand (FLT3L), and IL-5, which are the conditions defined for eosinophil differentiation. Progenitors from IRW mice were also found to be unable to differentiate into mast cells in the presence of IL-3 and SCF. The authors found that while the progenitor cells were unable to respond to factors *ex vivo*, OVA sensitization and challenge resulted in eosinophilia. This suggests that there are other endogenous compensatory mechanisms which drive differentiation, and this requires further elucidation (19).

## HSPC Differentiation into Mast Cells

The hematopoietic lineage for mast cell development is unique in that mast cells leave the bone marrow as progenitors rather than as circulating end-stage cells. Mast cell progenitors will accumulate in different body tissues under the influence of locally produced factors that determine the final phenotype of the mast cell. Mast cell progenitors in the periphery, are identified by their expression of FCεRI, c-Kit, CD13, CD33, CD34, CD38, and by their ability to form granulated MC colonies in culture (41, 42).

In order to analyze the role of the bone marrow microenvironment in human hematopoietic lineage development, a transgenic mouse strain was developed that expressed human membrane-bound SCF. CD34^+^ cells isolated from human cord blood were then transplanted into NOD/SCID/IL2rgKO immunocompromised mice that expressed membrane-bound SCF (hSCF Tg NSG). These transgenic mice expressing human SCF had rapid expansion of human CD45^+^ hematopoietic cells compared to non-transgenic NSG recipients. Since SCF-c-Kit signaling is critical for the maintenance of stem and progenitor cells, hSCF Tg NSG contained mast cells in the lung cellular infiltrates. Membrane-bound hSCF in the bone marrow resulted in enhanced development of CD33^+^ myeloid cells from the engrafted HSCs. Transgenic expression of human membrane-bound SCF influenced human myeloid development and mast cell development in hematopoietic organs and mucosal tissues along with the high chimerism of human hematopoietic cells in hematopoietic organs (20). Al-Mushen and co-investigators (43) report the recovery of SCF mRNA-positive cells from bronchial washings in allergic asthmatics at a higher number compared to normal controls. While bronchial biopsy showed that SCF expression was present primarily in the epithelium, it was determined that alveolar macrophages were found to be the major source of SCF in bronchial washings from asthmatic subjects. Since the receptor for SCF, c-kit, is found on HSPCs and not on monocytes, it is probable that these increased macrophages are differentiated from circulating HSPCs (43).

## Recruitment of Mast Cell Progenitors into Tissues

In order for mast cell progenitors to migrate to the lung, they must express α4β7 and α4β1 (VLA-4) integrins. These integrins will bind to CXCR2-regulated VCAM-1 on endothelial cells. OVA has been found to upregulate VCAM-1 expression suggesting that mast cells further propagate inflammatory signals in the asthmatic lung (44). There is evidence that mast cell recruitment into tissues may also involve SCF binding to c-Kit, resulting in the activation of the PI3K pathway. Thus it appears that SCF is a major chemotactic factor for mast cells (42, 45).

Another chemokine involved in the attraction of mast cells to the tissue is CCL2. In OVA-challenged mice, the levels of CCL2 and CXCL1 are increased in BAL fluid compared to non-sensitized mice. In freshly isolated bone marrow, the functionality of the mast cell progenitors to migrate to CCL2 was demonstrated *in vitro* and further illustrated at the *in vivo* level by using subepithelial irradiation and bone marrow adoptive transfer. Examination of the bone marrows indicate that CCR2^−/−^ OVA-challenged mice reconstituted with WT bone marrow have a significant reduction in the concentration of mast cell progenitors (46). This indicates that CCR2/CCL2 signaling is imperative to the recruitment of mast cell progenitors to the lung during antigen challenge and that this requires participation of stromal and bone marrow elements. This study also denotes that CCL2 in the bone marrow and CCR2 in the lung are important for mast cell progenitor trafficking in the allergic airway models used and further highlights the complex signaling mechanisms in mast cell progenitor trafficking.

## HSPC Differentiation into Monocytes/Macrophages

Macrophages are terminally differentiated tissue dwelling cells derived from circulating monocytes. Most tissue macrophages are derived from hematopoietic stem cells and their local expansion within tissues can be due to local proliferation of existing macrophages or due to infiltration of blood-derived monocytes. To fulfill many different roles in the tissue, macrophages can adapt different phenotypes based on signals they receive from their environment. Depending on their level of activation, macrophages can differentiate into M1 or M2 cells. M1 macrophages act as pro-inflammatory cells in host defense against intracellular pathogens and cellular debris. M1 macrophages are induced by Th1 cytokines, interferon-γ (IFN-γ) and tumor necrosis factor-α (TNF-α), and have been shown to be stimulated by GM-CSF and LPS. M2 macrophages are induced by Th2 cytokines, IL-4, and IL-13, and also influenced by IL-10 and M-CSF. M2 macrophages produce a wide range of factors that are involved in airway remodeling that either lead to restoration of the tissue or pathological fibrosis (47, 48 http://bloodjournal.hematologylibrary.org/content/79/4/846.full.pdf).

Monocytes/macrophages perform essential functions in homeostasis, infection, tissue repair, and resolution of inflammation. These cells have pleiotropic functions in the body in that they can interact with cells with progenitor or stem cell properties and that this interplay may contribute to repair and remodeling (49, 50). Re-establishment of tissue homeostasis in response to injury requires infiltration of inflammatory cells and activation of resident stem cells. Full tissue recovery requires that the inflammation is resolved.

## Recruitment of Monocyte/Macrophage Progenitors into Tissues

The factors that influence monocyte/macrophage trafficking to the lung during an inflammatory event are not well established. However, in inflammatory disease states of other tissues such as the liver, CCR2 activation on the surface of monocytes by monocyte chemoattractant protein 1 (MCP-1) and MCP-3 factors at the site of inflammation may be responsible for the homing of monocyte/macrophages to the area of injury (51). Therefore, mechanisms of monocyte/macrophage migration during airway inflammation deserve further attention.

There is evidence that both M1 and M2 cells are involved in asthma. M2 macrophages have been found to correlate with the severity of allergic airway inflammation in human and mice. M1 macrophages have been suggested to be beneficial to prevent allergic sensitization but may promote development of M2 macrophages in the presence of established disease (5). In OVA-challenged mice, an increase in macrophages were recovered in the BAL fluid following 1 week of OVA challenge, compared to saline control mice. This increase in macrophages following allergen challenge was also observed in macrophages recovered in the tissues. While the number of macrophages in BAL decreased following weekly exposures to OVA, there was an increase in tissue macrophages, further demonstrating macrophage involvement in the pathogenesis of asthma (52).

Macrophage proliferation and activation requires MAPK. MAPK activation requires phosphorylation on threonine and tyrosine residues that are located in the activation loop. MAPK activation are regulated by the dominant action of protein phosphatases, as evidenced by the fact that activation is reversible even in the continued presence of activating stimuli. A key regulator of macrophage proliferation and activation is mitogen-activated protein kinase phosphatase-1 (MKP-1). MKP-1 is a dual-specificity phosphoprotein phosphatase and has been identified as a negative regulatory factor of the innate immune system and as a key regulatory factor for macrophage proliferation and activation (49). Expression of MKP-1 in tissue repair following damage was demonstrated in muscle macrophages in which the tissue from MKP^−/−^ mouse lacked inflammatory cytokine expression despite persistent tissue damage unveiling a function for macrophage controlling stem cell dependent inflammation in damaged muscle tissue repair (53). Regulation of innate immune inflammation could thus be regulated by this phosphatase. Therefore, application of therapeutics based on MKP-1 modulation could decrease asthma morbidity and intensity.

Signals released from M1 and M2 macrophages induced the migration of meso-angioblasts in a chemotactic assay (54). The results of this study support that macrophage recruit stem cells to the site of injury in the tissue and the ability of precursor cells to reconstitute the damaged tissue depends on the signals generated *in situ* by the macrophages. Therefore, intervention to allergic disorders could be targeted in tempering stem cell migration by modulating macrophage release of inflammatory cytokines. However, the origin of release of these chemoattractants is still poorly characterized (54).

## Lymphocytes

Lymphocytes, such as T and B cells, differentiate from a common lymphoid progenitor and increased leukocyte progenitors have been found in asthma patient blood (55). Th17 cells mediate tissue inflammation and autoimmunity. TGF-β and IL-6 induce Th17 differentiation from naïve CD4^+^ cells through regulation of chromatin remodeling and IL-23 is required for expansion and maintenance of Th17 cells. RORγt is considered to be a key transcription factor for Th17 cells because its expression is induced specifically during differentiation of Th17 cells by TGF and IL-6. However, some cells that express RORγt function as T-regulatory (Treg) cells. This raises the question of the role of RORγt in the early differentiation process of T cells from HSC. In RORγt-BMT mice where RORγt expression is forced at the HSC level, increased expression of both Th17 and Treg expression was found. A series of *in vivo* experiments in which HSCs expressing RORγt were transplanted into lethally irradiated mice, the effects of RORγt on T cell development was evaluated. In contrast to mice transplanted with HSCs expressing IL-17 which died within 14 days after transplantation, those with reconstituted bone marrow hematopoiesis survived without any autoimmune disorders during the observational period of over 16 weeks even though they had an increased number of Th17 cells showing intracellular expression of IL-17. Mice with increased number of Tregs had attenuated immune response when challenged in a chronic hypersensitivity assay. This phenomenon was supported with adoptive cell transfer of CD4^+^ T cells from these mice to recipient mice. Thus, these experiments suggest that the surrounding cytokines, such as IL-5 and TGF-β, influence the differentiation of Th17 to Treg cells by influencing the expression of RORγt which ultimately results in decreased tissue damage (56).

A protease responsible for cell migration and is highly expressed in the asthmatic lung is a disintegrin and metalloprotease domain 8 (ADAM8). Adoptive transfer experiments showed that ADAM8 on hematopoietic as well as on non-hematopoietic cells was required for full asthmatic response, ADAM8-deficient T-lymphocytes significantly decreased the asthmatic response. Thus, this enzyme may also be a potential target for the inhibition of inflammatory response in asthma (57).

## Fibrocytes

In addition to differentiation into eosinophils, basophils, and mast cells, CD34^+^CD45^+^ cells can differentiate into several types of tissue dwelling cells involved in the remodeling of the airway structure (9). Fibrocytes are thought to be derived from monocytes that have similar features to that of fibroblasts and macrophages (58). Fibrocytes express different fibroblast proteins such as vimentin, collagen I and III, fibronectin, as well as CD34 and CD45 (59). During the wound-healing process, the initially expressed CD34 will decrease with the induction of fibrocytes being induced by TGF-β1 and endothelin-1 (ET-1). Evidence for fibrocytes in asthma was observed in patients with chronic obstructive asthma. Here, patients with chronic obstructive asthma compared with asthmatic patients with normal pulmonary function and healthy subjects had significant increase in the percentage of circulating fibrocytes, as defined on the basis of expression of CD34^+^, CD45^+^, and collagen I^+^ cells in non-adherent non-T cells (60).

Bone marrow-derived stem cells have been found to be responsible for the proliferative cells in the asthmatic airway during airway remodeling. In GFP^+^ bone marrow chimera mice, chronic airway inflammation is characterized by increased thickness of the airway subepithelial basement membrane and smooth muscle layers. GFP^+^ bone marrow chimera cells produce collagen I and α-smooth muscle actin in OVA-sensitized and challenged mice compared to control mice (61). Bone marrow-derived fibrocytes was further defined from fibroblasts isolated from cockroach antigen-challenged mice. Cockroach antigen-challenged GFP^+^ bone marrow mice had increased fibroblasts in the lung compared to healthy mice. Lung fibroblasts cells also express telomerase reverse transcriptase (TERT), which is induced in lung injury and fibrosis. The 70% of the TERT^+^ cells were found to be mostly derived from bone marrow (62). Therefore, bone marrow-derived adult stem cells can contribute to the increased fibroblast population found in respiratory disease and are involved in the pathogenesis of airway remodeling in asthma.

## Therapeutic Considerations

Given the involvement of hematopoietic progenitor cells in mediating allergic inflammation and airway remodeling in asthma, various molecules involved in the molecular process of the differentiation of hematopoietic progenitor cells offer attractive therapeutic targets in alleviating asthma. Therapies for asthma still strongly rely on inhaled corticosteroids and long-acting inhaled β_2_ agonists. However, these treatments may not be able to fully reverse the effects of inflammation and airway remodeling. Newer therapeutic approaches include treatment with IL-5 antibody. This was found to decrease CD34^+^/IL-5Ra cells in atopic patients. Anti-IL-5 treatment may regulate local tissue infiltration of eosinophils (10, 63). However, so far this has not been found clinically successful.

Another target is the regulation of p38 MAPK. MKP-1 can switch off p38 signaling and cytokine production in monocytes/macrophages. This action was found to be increased in the presence of vitamin D (64). This also gives further evidence in offering vitamin D supplementation as a treatment option in vitamin D. Reduced vitamin D levels are associated with impaired lung function, increased AHR, and reduced glucocorticoid response. MKP-1 was found to be increased in the lung with higher vitamin D levels (65).

Oligonucleotides are an emerging family of drugs with potential to treat asthma. An inhaled anti-sense drug, TPI ASM8, contains two modified phosphorothioate anti-sense oligonucleotides (AON), one targeting the common beta chain (βc) of the IL-3/IL-5/GM-CSF receptors and the other targeting the chemokine receptor CCR3. Inhalation of TPI ASM8 significantly improves lung function and sputum eosinophilia after allergen inhalation challenge in mild asthmatic subjects with early and late asthmatic responses. Thus, TPI ASM8 may effectively block accumulation of eosinophils and eosinophil progenitors in the airways following allergen challenge (66).

## Conclusion

**Figure 2:**
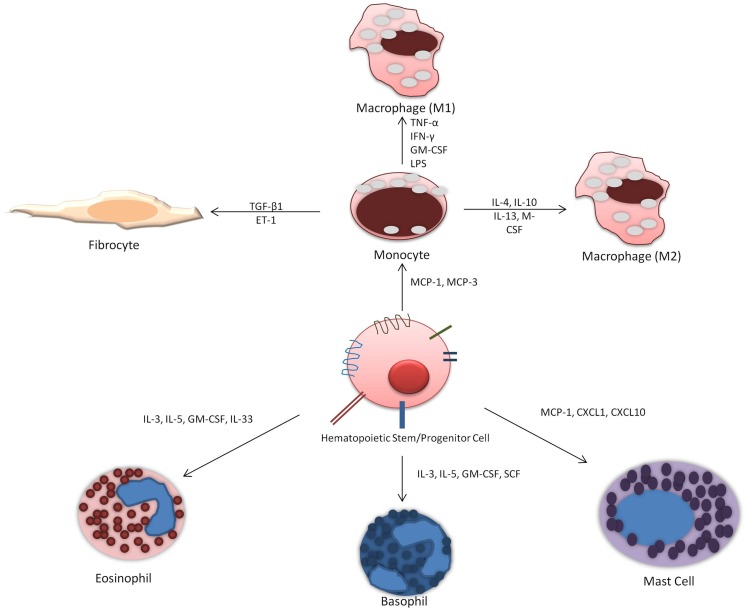
**Differentiation of hematopoietic progenitor cells into immune cells: HSPCs can potentially differentiate into distinct immune cells *in situ* depending on the presence of locally elevated factors and cytokines**.

Allergic asthma is a complex disease characterized by chronic airway inflammation and increased airway hyperresponsiveness as a reaction to allergen stimuli. There is evidence to support that hematopoietic stem cell homing to the site of inflammation release cytokines to influence and exacerbate allergic inflammation and are also influenced by local cytokines and growth factors at the site of injury. Their ability to differentiate *in situ* into different immune cells is dependent on cytokine and growth factors (Figure 2). Current investigative approaches looking at HSPCs differentiation potential primarily focus on eosinophil and basophil progenitors. However, important role of monocytes and mast cells should not be overlooked and the potential for differentiation into fibrocytes must also be considered. Future studies should address the ability of HSPCs to differentiate *in situ* as well as focus on factors that release HSPCs from the bone marrow and when this occurs. Therefore, the management of hematopoietic progenitor cells differentiation offers a potential therapeutic target. Therapy that targets the maturation of CD34^+^ HSPCs specifically into the inflammatory cells in the lung could result in reduced tissue maturation of hematopoietic cells and thus decreased allergic airway inflammation.

## Conflict of Interest Statement

The authors declare that the research was conducted in the absence of any commercial or financial relationships that could be construed as a potential conflict of interest.
